# Species diversity of mosquitoes of the Genus *Culex* (Diptera, Culicidae) in the coastal areas of the Persian Gulf

**DOI:** 10.3934/publichealth.2019.2.99

**Published:** 2019-03-26

**Authors:** Mehdi Khoobdel, Davoud Keshavarzi, Seyed Hassan Mossa-Kazemi, Hossein Sobati

**Affiliations:** 1 Health Research Center, Life style institute, Baqiyatallah University of Medical Sciences, Tehran, Iran; 2 Department of Medical Entomology and Vector Control, School of Public Health, Tehran University of Medical Sciences, Tehran, Iran

**Keywords:** *Culex*, species diversity, mosquitoes, vector control

## Abstract

**Background:**

Lack of information about the dispersal of vector species barricade surveillance and control.

**Aims:**

Therefore, the aim of the present study was to determine the species diversity of *Culex* mosquito's larvae in the coastal areas of the Persian Gulf.

**Methods:**

Mosquito larvae were collected from six places in three main environmental categories: urban (UA), rural (RA) and uninhabited areas (UNA), using dipping technique. Four dips were taken from each breeding site (350 ml each). Larval investigation was conducted two times a month during the study period. Diversity studies were conducted separately for each category by calculating classic diversity indices.

**Results:**

In total, 1369 specimens belonging to 10 different species were collected and identified, as follows: *Culex hortensis*, *Cx. laticinctus*, *Cx. mimeticus*, *Cx. perexiguus*, *Cx. pipiens*, *Cx. modestus*, *Cx. sinaiticus*, *Cx. theileri, Cx. torrentium* and *Cx. tritaeniorhynchus*. None of these mosquito species have been recorded previously in this region. Diversity analysis indicated higher species richness for RA (*Margalef* 1/26). The average diversity indices for the three environment types ranged from 1.50 to 1.64 for Shannon index and from 0.730 to 0.738 for Simpson index.

**Conclusions:**

Biodiversity analysis indicated that species diversity in rural, urban and uninhabited areas is somewhat similar. Therefore, attention to all areas in vector control programs is essential.

## 1. Introduction

Mosquitoes are the most important vectors of public health due to their roles in the transmission of diseases such as Malaria, Lymphatic filariasis, West Nile fever (WNV)‚ Zika, Sindbis fever‚ Dengue fever‚ and Encephalitis [1]–[4]. Among these mosquito-borne diseases‚ Malaria‚ West Nile‚ Sindbis‚ Dengue fever, and Dirofilariasis have been reported in Iran [2],[3],[5],[6]. According to a new study, the risk of Rift Valley fever in Iran is remarkable [7]. In addition, Zika and Dengue fever threatening the Persian Gulf region and southern Iran [8],[9] *Dirofilaria immitis* (causes heartworm disease) has been reported from Fars province [10]. In Iran, natural infection with third stage of *D. immitis* larvae was recorted in *Culex* *theileri*, this could be the main vector of this roundworm parasite [3]. Sero-survey of WNV in the equine populations in Iran indicated circulation of virus in southwestern provinces [11]. *Culex pipiens* reported as a bridge vector for WNV [12]. Therefore, due to the possibility of culicid mosquitoes to transmit vector-borne diseases, the necessity of the present study is highlighted. There is an urgent need to gather baseline data on species richness and diversity of mosquitoes so that their roles as vectors of various human and animal diseases may be better understood [13]. There is no information about the fauna and diversity of mosquitoes in the study area, but some species of mosquitoes including *Cx. perexiguus*, *Cx. quinquefasciatus*, *Cx. sitiens*, *Cx. arbieeni*, *Cx. tritaeniorhynchus*, *Culiseta longiareolata* and *Ochlerotatus caspius* have been recorded in south of Iran and Iranian islands in the Persian Gulf [2],[9]. Therefore, the main objective of the present study was to determine species diversity of medically important mosquitoes (Diptera, Culicini) in the coastal areas of the Persian Gulf.

## 2. Materials and methods

### 2.1. Study area

To develop the study, six sites were selected based on ease of access and topography in the different areas (urban areas (UA), rural areas (RA) and uninhabited area (no human populations) (UNA)) of the north of Bushehr province (50°31′1E, 29°34′45N), in the Southwest of Iran. Date palm and shrimp cultivation are two of the most important cultural activities in rural areas. Two sites for each of those areas were selected. During this period of study (January–December 2017), the average maximum and minimum temperatures were 48 °C and 1 °C in July and February, respectively (Table 1).

**Table 1.** Ambient temperature and total rainfall in the north of Bushehr, January–December 2017.

Months	Jan	Feb	Mar	Apr	May	Jun	Jul	Aug	Sep	Oct	Nov	Dec
Minimum temperature (°C)	5	1	10	14	22	24	28	27	24	15	10	5
Maximum temperature	27	26	30	38	45	47	48	46	42	39	34	30
Mean temperature	16	15	21.4	28.7	33.9	36.2	36.8	37.1	34.7	30.6	22.5	17.8
Total rainfall (mm)	8.2	29.2	28.8	0.1	5	0	0	0	0	0	34.2	2

### 2.2. Sampling methods and taxonomic identification

In order to study the ecology of mosquitoes, sampling was carried out by dipping technique with a metal dipper for collecting larvae. Four dips were taken from each breeding site (350 ml each). Larval investigation was conducted two times a month during the study period.

All the samples were brought to the laboratory of the Entomology Department, Tehran University of Medical Sciences, Iran. The mosquito larvae were preserved in 75% ethanol and the microscopic slides were prepared using the chloral gum mounting. Microscope was used for the taxonomic study and identification, up to the species level using taxonomic keys available in literature [14].

### 2.3. Biodiversity and statistical analysis

Diversity studies (alpha diversity) were conducted separately for each category (rural, urban and uninhabited areas) by calculating classic diversity indices like Margalef's (S − 1)/lnN [S = total number of species and N = total number of individuals) and Simpson's indexes (λ = 1 − ∑pi^2^, where pi = ni/N [ni is number of individuals of taxon i]) [15]. Dominance = 1 − Simpson index. Ranges from 0 (all taxa are equally present) to 1 (one taxon dominates the community completely). Shannon diversity index (H′ = −[Σ(pi lnpi)]) is commonly used to characterize species diversity in a community, accounting for both abundance and evenness of the species present [15]. Equitability index measures the evenness with which individuals are divided among the taxa present.

Two indices (Jaccard's and Whittaker's indices) were used for similarity and dissimilarity between habitats [2],[15]. Data were analyzed using PAST software version 3.14 (Paleontological Statistics Software Package). Non-parametric tests were used to evaluate differences in species abundances and weather-related variables between months. Because of our data were not normally distributed. The Pearson's correlation test was used to measure of the strength of the association between the variables.

## 3. Results

**Figure 1.**
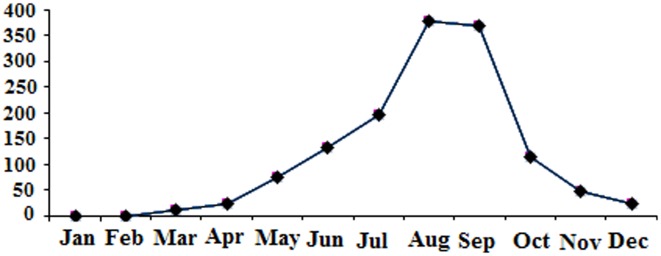
Monthly variation in abundance of all mosquito species collected in all areas of the study, January–December 2017.

**Figure 2.**
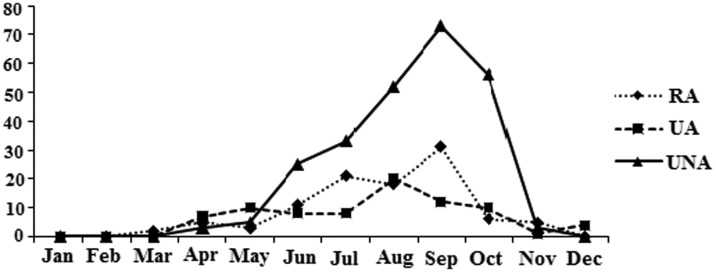
Monthly abundance of dominant mosquito species (*Cx. theileri)* at three habitat types (RA; rural areas, UA; urban areas, UNA; uninhabited area).

A total of 1369 specimens belonging to 10 different mosquito species were collected and identified. These were: *Cx. hortensis* Ficalbi, *Cx. laticinctus* Edwards, *Cx. mimeticus* Noe, *Cx. perexiguus* Theobald, *Cx. pipiens* Linnaeus, *Cx. modestus* Ficalbi, *Cx. sinaiticus* Kirkpatrick, *Cx. theileri* Theobald *Cx. torrentium* Martini and *Cx. tritaeniorhynchus* Giles. In the dipping collection, *Cx. theileri* (28.0%), *Cx. pipiens* (26.1%), *Cx. tritaeniorhynchus* (23.1%) were predominated, respectively. The greatest number of mosquitoes were collected from RA (723 specimens) and the lowest in the UA (209 specimens) (Table 2). Monthly variation in abundance of mosquitoes is provided (Figure 1). The highest number of mosquitoes were collected in August (379) and September (370 specimens), while the lowest were in January (0 specimen) and February (3 specimens). Depending on the month, difference was not significant in mosquito abundances according to Kruskal-Wallis test (*P* = 0.44). Analysis with Kruskal-Wallis and Mann-Whitney tests showed that, difference between environments (UA, RA and UNA) and abundance of mosquitoes was not significant (*P* = 0.33). Monthly abundance of dominant mosquito species (*Cx. theileri*) at three habitat types is also provided (Figure 2). Overall, in the present study there was a significant positive relationships between mean temperatures and abundance of mosquitoes (r = 0.75, *P* = 0.005). In contrast, abundance of mosquitoes decreased with increasing precipitation (r = −0.42, *P* = 0.16).

**Figure 3.**
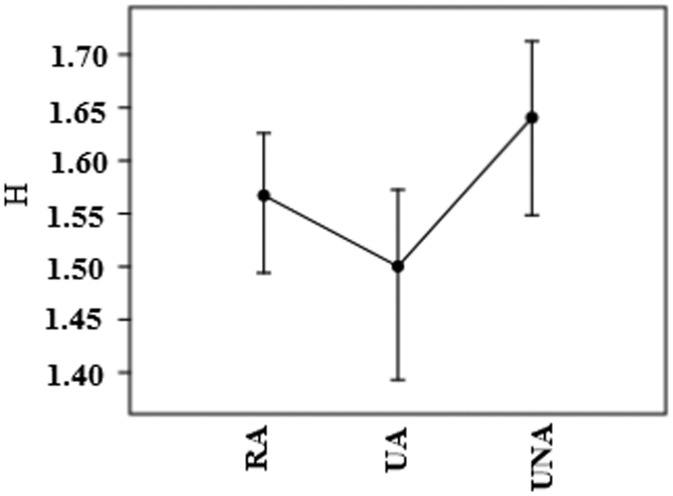
Shannon index for each environmental category (UA, RA and UNA).

A greater species richness was found in RA (M = 1/21), while UA has the lowest (M = 0.93). The average diversity indices for the three environment types ranged from 1.50 to 1.64 for Shannon index and from 0.730 to 0.738 for Simpson index. Shannon index was highest in UNA and lowest in UA. While, Simpson index in UA was highest (Figure 3). The T-test showed no statistically significant difference between Shannon index (t = 1.17, *p* = 0.24) and Simpson index (t = 0.39, *p* = 0.69) in RA and UA. Similarly, there was also no statistically significant difference between Shannon index (t = −1.36, *p* = 0.17) and Simpson index (t = 0.20, *p* = 0.83) in RA and UNA. But, there was a significant difference between Shannon index in UNA and UA (t = −2.26, *p* = 0.024). However, difference between Simpson index was not significant in these areas (t = −0.15, *p* = 0.87). Greater evenness was observed in UA, because the most dominant species do not show such a strong influence as in the two other environments. Where, *Cx. theileri* in UNA and *Cx. pipiens*, *Cx. theileri* and *Cx. tritaeniorhynchus* in RA were dominant and shows a strong influence.

**Table 2.** Composition and localities of the culicini mosquitoes collected in the north of Bushehr, January–December 2017 (RA; rural areas, UA; urban areas, UNA; uninhabited area).

Species	RA	UA	UNA	Total (%)
*Cx. theileri*	104	80	200	384 (28.0%)
*Cx. pipiens*	283	30	44	357 (26.1%)
*Cx. tritaeniorhynchus*	215	58	43	316 (23.1%)
*Cx. modestus*	8	25	-	33 (2.4%)
*Cx. sinaiticus*	19	-	36	55 (4.0%)
*Cx. perexiguus*	48	-	68	116 (8.5%)
*Cx. mimeticus*	28	11	26	65 (4.7%)
*Cx. hortensis*	-	-	17	17 (1.2%)
*Cx. laticinctus*	8	-	-	8 (0.6%)
*Cx. torrentium*	10	5	3	18 (1.3%)

## 4. Discussion

In the present investigation, 10 species of mosquitoes were identified. Among them there are some major vectors of human and animal diseases such as *Cx. pipiens* (Usutu virus, WNV), *Cx. theileri* (Dirofilariasis, Rift Valley fever), *Cx. perexiguus* (Sindbis fever, West Nile fever), *Cx. tritaeniorhynchus* (Japanese encephalitis, Rift Valley fever, Sindbis fever, West Nile fever) [3],[9],[16].

*Culex theileri* (28.0%) was appeared from March to December to make a peak with 135 individuals in September, when the mean temperature was 34.7 °C. According to a study in Fars Province, *Cx. theileri* reached its peak in June (n = 311) [17].This finding suggests that the oviposition activity of *Cx. theileri* is limited by the temperatures. It was the most abundant species in urban areas, this is in disagreement with its dispersal on the western region of Spain [18]. Monthly activities of this species in Mohr and Darab counties (Fars Province) were reported from April to September [2]. The present study does not provides similar findings.

*Culex theileri* is found in the most parts of the world. This mosquito has been recorded in all provinces of Iran and has involvement in the transmission of *Dirofilaria immitis* nematode [3]. In the study of ecology of mosquitoes in Fars, *Cx. theileri* preferred habitats with permanent water and without plants [17]. This finding is not similar to the present study.

*Culex pipiens* was the most frequent species in rural areas, whereas *Cx. tritaeniorhynchus* came in the second order. This is in disagreement with its dispersal on the western region of Spain, where, it was the most abundant species in in urban environments [18].

According to Margalef index, RA show the highest diversity. The reason for this might be due to ecological and climatological factors. In the present study, Shannon and equitability indices shows that in RA, species such as *Cx. tritaeniorhynchus* and *Cx. pipiens* intensely dominate the rest of species present in the community. Something similar occurs in the case of UNA, where *Cx. theileri* develop a strong influence. Finally, greater evenness degree can be observed in UA, because the most dominant species do not show such a strong influence as in the two other cases. There is very little data on the biodiversity in mosquito populations in Iran. In the present study, the maximum value for Shannon's index was 1.64. According to a study in Brazil, the maximum Shannon's index was 2.16 [19]. In another research in Iran, the maximum value for Shannon's index was 1.7 [2]. Normally, the Shannon index in real ecological units ranges between 1.5 and 3.5, therefore, a smaller amount makes it difficult to interpret actual species diversity [20].

We think that these differences in previous reports are due to the differences in the climate, sample size and data analysis method. Diversity indices are different from one season to another, consequently a diversity index result needs to be compared to other results of the same area [19]. Biodiversity studies have been ignored in this area, so comparison of results in the future should be made more precise.

## 5. Conclusion

In our research, some possible vectors of medical and veterinary importance were identified. In this area of study, mosquitoes were more active in August and September. Biodiversity analysis indicated that species diversity in rural, urban and uninhabited areas is somewhat similar. Therefore, attention to all areas in vector control programs is essential.
